# Case study guided development of an implementation science framework and checklist for campus sexual violence intervention

**DOI:** 10.1186/s44263-025-00215-0

**Published:** 2025-11-25

**Authors:** Rebecca Fielding-Miller, Anh Vo, Vinton Omaleki, Pinky Mahlangu, Yandisa Sikweyiya, Fortunate Shabalala, Sakhile Masuku, Menelisi T. T. Dlamini, Mercilene Machisa

**Affiliations:** 1 Herbert Wertheim School of Public Health and Human Longevity Science, University of California, San Diego, USA; 2 Division of Infectious Diseases and Global Public Health, School of Medicine, University of California, San Diego, USA; 3 South African Medical Research Council, Cape Town, South Africa; 4 Department of Community Health Nursing, University of Eswatini, Kwaluseni, Eswatini

**Keywords:** Campus sexual violence, Implementation science, Translational research, Global health, Gender-based violence

## Abstract

**Background:**

Campus sexual violence interventions must be scalable and effective in real-world settings. However, campus sexual violence interventions have unique implementation contexts that are not fully addressed by the most common implementation science (IS) frameworks. Hybrid studies—designs that incorporate effectiveness and implementation outcomes into a single trial—are an important tool to shorten the translation pipeline and enhance the efficacy and effectiveness of these interventions. We conducted a series of case studies to explore the applicability of the Consolidated Framework for Implementation Research (CFIR) to campus sexual violence interventions and to develop an adapted framework and checklist that could be used to advance the IS of campus sexual violence intervention research.

**Methods:**

We developed four case studies of campus sexual violence interventions from the United States, South Africa, and Eswatini. Two were delivered digitally (United States and Eswatini) and two were delivered in person (South Africa and Eswatini). We analyzed these cases using a comparative design guided by CFIR’s theorized constructs, aiming to identify IS concerns that are likely unique to campus sexual violence implementation (CSVI) research. These analyses were used to develop a CSVI-specific IS checklist and planning worksheet.

**Results:**

We identified multiple cross-cutting issues unique to the IS of campus sexual violence interventions: policy and legal framework, team praxis, relationships, context, infrastructure, and people. We situated these within a conceptual framework to reflect their intersections and developed a checklist of considerations at the planning, implementation, and post-project reflection phases.

**Conclusions:**

The field of campus sexual violence intervention research will benefit from the integration of IS methodologies. While we found CFIR to be a useful starting point, it requires certain key modifications to fully support sexual violence intervention research. The adapted CFIR framework we developed from a cross-national set of case studies offers a valuable tool to meet the needs of funders, researchers, and practitioners in the global north and south.

**Supplementary Information:**

The online version contains supplementary material available at 10.1186/s44263-025-00215-0.

## Background

The evidence on effective strategies to prevent campus sexual violence (CSV) in the global South is still nascent. Globally, few evidence-based interventions (EBIs) have been shown to effectively reduce the perpetration of or experiences of violence among university students [1–3]. However, the evidence base is growing (as demonstrated by the special issue in which this manuscript appears). It is crucial that researchers and practitioners are prepared not only to disseminate and implement EBIs at scale, but to ensure that these interventions continue to be effective once they are in widespread use.

Sexual violence (SV) is a complex phenomenon driven by individual, relational, community, and societal factors [2]. Effective interventions must account for each of these levels as well as how they intersect [4]. Even early-stage CSV intervention (CSVI) pilot projects take place within complex systems where academic and social culture, priorities, funding, and policies can vary significantly within individual campuses and across institutions. Implementation scientists have argued that traditional effectiveness trials, which assess an intervention’s internal validity under ideal conditions, may not be appropriate starting points for community-based interventions that are highly context-specific [5]. This is likely to be especially true for CSVI research, which takes place in institutions of higher education that are extremely variable and require specific tailoring to meet local needs and incorporate stakeholder interests.

Most CSVI research has historically taken place in the global north, and the few EBIs that do exist elsewhere were created in the global north and later adapted for low- and middle-income countries (LMIC) [2]. A 2024 review identified 31 studies published between 2005 and 2023 that sought to assess barriers and facilitators to interpersonal violence interventions. None of the reviewed studies assessed implementation concerns for interventions in higher education settings or interventions in the global South [6]. The few studies conducted in southern Africa identified gender inequality, high rates of poverty and unemployment (a legacy of colonial extraction), food insecurity, and heightened HIV risk as factors contributing to distinct needs for CSVI programming. These factors are compounded by power imbalances in relationships and emotional barriers to assertiveness. Institutionally, weak policy enforcement and mistrust in support services can lead to under-reporting and low use of survivor resources [7, 8].

Notably, very few of the interventions identified in the 2024 review were evidence-based and/or informed, and this lack of rigorous evidence constituted a major implementation barrier in many settings [6]. Additionally, funding constraints—particularly for research and effectiveness trials in the global South—further impede the development, evaluation, and scaling of EBIs, limiting their availability and adaptation in these contexts [9]. However, the translational lag from intervention development to effectiveness evaluation to successful dissemination and scale-up is not unique to CSVI research. Evidence suggests that biomedical interventions take an average of 17 years to travel the same pathway [10]. However, the scale and urgency of CSV calls for significantly accelerating the pipeline from conceptualization to pilot, effectiveness studies, and onwards to implementation at scale.

Implementation science (IS) frameworks are essential for translating research into practice by identifying best practices for implementing interventions in ways that maintain program effectiveness [11]. They provide structured approaches for planning, executing, and evaluating efforts, enhance stakeholder communication, and guide researchers in selecting strategies tailored to diverse contexts. IS frameworks bridge the research-to-practice gap by informing the optimization of intervention protocols, delivery methods, and cost-effectiveness for large-scale dissemination. Utilizing and advancing IS for CSVI work is a crucial step forward, given the context-specific and multi-level nature of these interventions.

Hybrid designs have emerged over the past decade to address some of these issues [12]. These designs incorporate both effectiveness and implementation outcomes to address the challenge of demonstrating an intervention’s effectiveness when “perfect conditions” are not only impractical, but likely to be so dramatically different from real world conditions that any findings produced in the research would be questionable [5, 13]. Hybrid designs exist along a continuum: type 1 hybrid designs heavily emphasize effectiveness evaluation with less emphasis on understanding potential barriers and facilitators should the intervention show promise, while type 3 hybrid designs primarily focus on establishing best practices for successful implementation at scale with a secondary focus on continuing to monitor indicators of intervention effectiveness [12].

The Consolidated Framework for Implementation Research (CFIR) is frequently used in IS studies to systematically consider potential barriers and facilitators to implementing and scaling up EBIs [14]. CFIR is the most commonly used IS framework for understanding barriers and facilitators to successful violence intervention implementation. The framework assesses the likelihood of implementation success across 5 domains: inner setting, outer setting, individual, innovation, and processes. CFIR has been utilized to assess implementation success across a wide range of public health topics and in high- and low-income settings [15]. However, some researchers have critiqued it as overly broad, noting its blind spots related to issues of power dynamics and historical injustices [16].

The objective of the current study was to develop a CFIR-informed IS checklist and framework that could be used to inform future CSVI research and implementation projects.

## Methods

We explored our research question using a multiple-case design, treating each case as a holistic phenomenon [17]. While there is some debate about whether case studies should be considered as one method in the broader qualitative methodology toolbox or as a unique methodology in their own right, case study designs are broadly ideal for research questions which seek to understand real-world, contemporary phenomena, especially when the boundary between a phenomenon and its context are not clearly defined [18–20]. Case studies typically fall into one of three categories, although the boundaries between these categories can be porous: *intrinsic* studies, which explore cases that are unique phenomena; *instrumental* case studies that use a single example as an illustration of broader principles; and *collective* case studies which analyze multiple exemplar cases to derive broader principles [17]. In all types, case boundaries are pre-defined by the research team, with specifics depending on the nature of the research question and theoretical framework (if any). Cases are purposively selected based on two broad principles: (a) their utility for answering the research question, and (b) accessibility and familiarity (i.e., can the research team access sufficient data to meaningfully describe the case, and are stakeholders sufficiently invested in potential study findings to allow access?) [17]. They typically triangulate across multiple forms of data to enhance credibility and confirmability [21], with attention to the ability to compare across multiple cases in collective designs [17].

We sought to address our research question by comparing 4 distinct cases of CSVI research. We used the CFIR tool as a framing device to bound our data collection, followed by inductive cross-case analysis to derive a CSVI-specific IS framework and checklist. We assessed our cases using a critical post-positivist lens that centered the question of power and power dynamics at each step [16, 17]. This power-critical lens is crucial for gender-based violence (GBV) research that aims to incorporate ‘empowerment’ components [22].

### Positionality

This project originated as informal conversations between colleagues who have led CSVI research in the United States and sub-Saharan Africa. The authors of this study comprise CSVI investigators, research staff, and student advisory board (SAB) leadership from South Africa, Eswatini, and the United States. M.M is a female, Black African social epidemiologist trained in quantitative and qualitative methods whose focus has been conducting GBV and mental health research and intervention development in Southern African countries. PM is a public health scientist with expertise in qualitative research and intervention co-development and evaluation. Her research interests are GBV, mental health, and HIV, focusing on adolescents, girls and young women, including gender diverse populations. YS is a public health researcher with expertise in three interrelated yet distinct research areas: gender identities, GBV, and social aspects of HIV. He has conducted qualitative and quantitative research on these topics in South Africa and other LMIC. FS is a medical anthropologist with experience in qualitative research. She has been involved with numerous campus sexual assault prevention research and practice projects in Eswatini as a co-investigator and a local advisor. FS led the development of the first sexual harassment policy at the University of Eswatini (UNESWA). SM is a senior lecturer at UNESWA, where she has served as a co-investigator and local advisor on campus sexual harassment prevention programming and research. She was part of the core team that developed UNESWA’s first sexual harassment policy. MTTD is an African young woman and a qualified nurse-midwife. She is the co-founder and chairperson of the Student Network Against Sexual Harassment (SNASH), a student initiative club that focuses on raising awareness against and educating students about sexual harassment to combat rape culture among students. RFM is a White woman and United States citizen. She has lived and worked in South Africa and Eswatini since 2006, including 2 years in rural South Africa and 2 years in Eswatini. She is a social epidemiologist with mixed-methods training. AVV is U.S citizen from a Vietnamese immigrant household who primarily resided, was educated, and works in the U.S. As a staff researcher and master-level graduate student, she analyzed data for Intimacy and Sexual Health Intervention Pilot for University Students (ISHIPUS) and worked closely with undergraduate students at UNESWA to coordinate the development of YMNN. RFM is her supervisor. VO is a White male upper middle class American staff researcher with mixed-methods training. He worked as a technical advisor for the YMNN project and a data manager for ISHIPUS.

### Case selection and definition

Our case selection was driven by our familiarity with a project and our ability to fully describe each case by the criteria described below. Our phenomenon of interest was research projects that assessed the effectiveness, feasibility, or acceptability of an intervention on SV among college or university students. We expanded our pool of potential cases by inviting colleagues who had also led CSVI research projects in the region, resulting in a final set of 4 cases.

We used the CFIR tool to conceptually bound our cases. We considered each case to be made up of the innovation (intervention which the research project was evaluating), outer setting or context in which the research project took place, inner setting (host study site and/or research team institutions), and individuals who participated in the project as stakeholders, researchers, study staff, study participants, or other interested parties. The temporal boundaries of a case also followed CFIR – we considered a case to begin at the point teaming—project conceptualization between collaborators and stakeholders—and to end with dissemination of findings and post-project team reflections.

Our holistic case definition encompassing both the phenomenon and its context also included:

1. The intervention itself and its pre-existing evidence base
2. Intervention site establishment (i.e., finding a partner campus)
3. Intervention adaptation or tailoring to the new content (if any)
4. Intervention programming logistics
5. Research logistics across the study lifespan (i.e., conceptualization, funding, data collection, dissemination)

### Cases

**Fig. 1:**
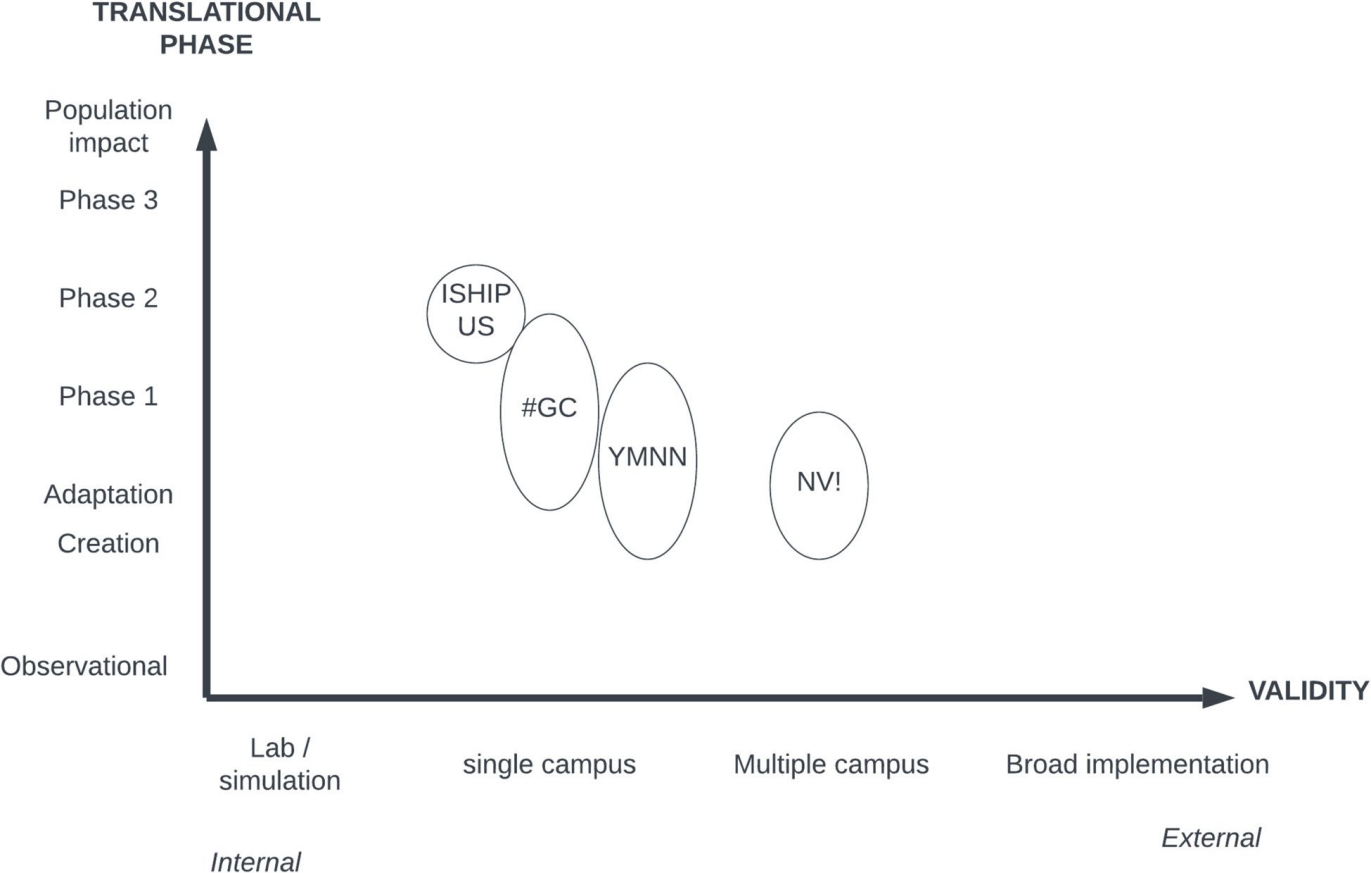
Case studies placement by focus on translational phase and internal/external validity focus

We identified 4 case studies which we felt represented a range of typical scenarios. One took place in the United States, one in South Africa, and two in Eswatini. Two were delivered online and two were delivered in person. Figure 1 shows where each study fell along two axes. The Y axis considers the study’s placement along the translational research pipeline across a spectrum of intervention development, adaptation, phases 1/2/3 of clinical research, to evaluation of population impact [23]. The X axis considers the study’s relative focus on internal or external validity, considering the variability and amount of control the research team was able to exert over implementation conditions [5].

### Data collection

After identifying our cases we created a dataset using an iterative process. First, those of us who had led an intervention as a PI or project coordinator wrote a brief description (vignette) of the case as bounded by our CFIR-informed case definition and with attention to the issues that inspired our initial research question. Vignette development was supported through references to handbooks and facilitator training guides developed for each project (e.g., Supplementary material 1), formative data collected during the development and/or evaluation of each intervention, reviewing grant applications (and their iterations over time until funded, as applicable), and study administrative records (e.g., emails and meeting notes).

**Fig. 2:**
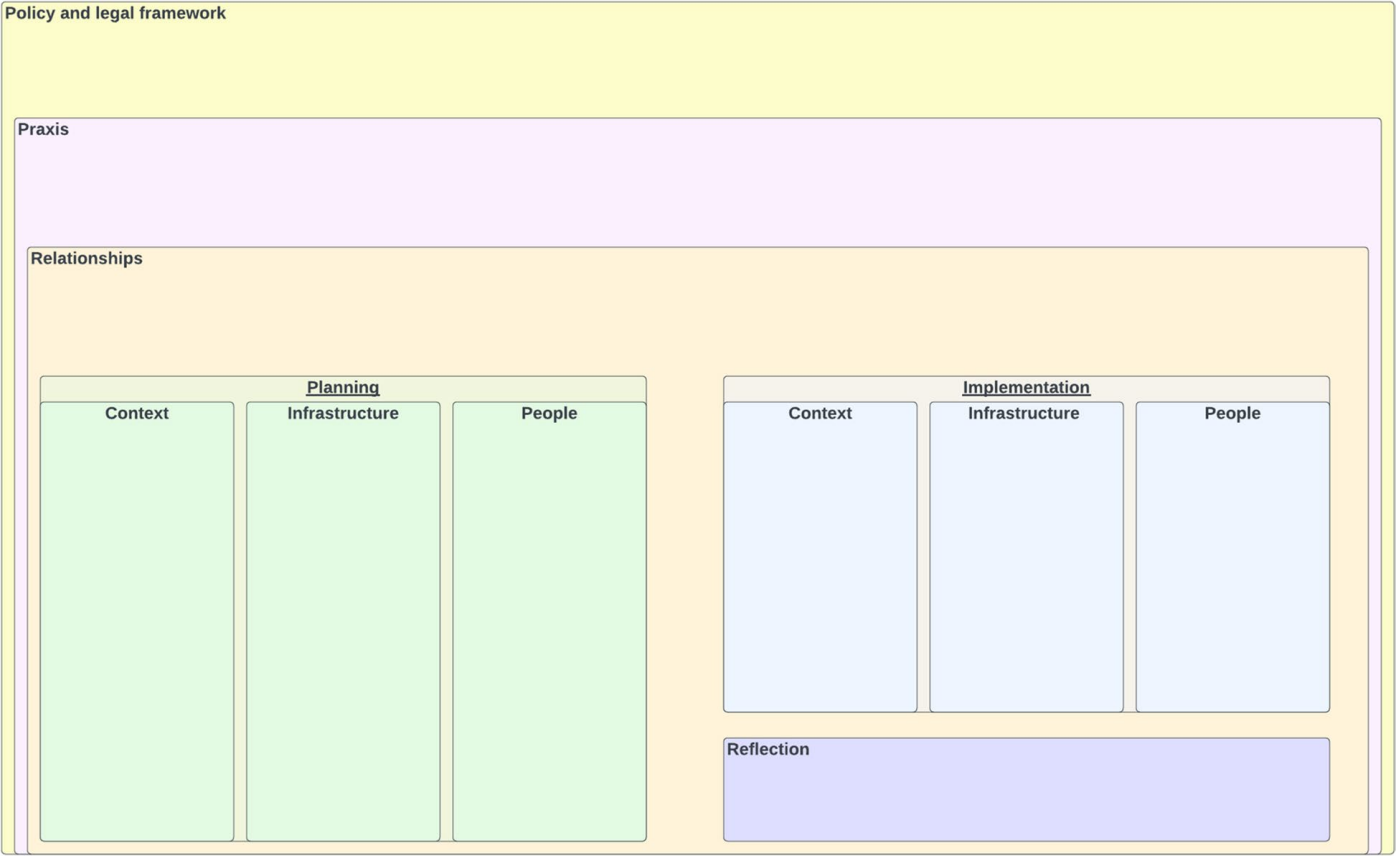
Conceptual framework and planning worksheet

After developing the four case vignettes we used the CFIR tool as an inductive coding scheme to create an analytic matrix, ensuring that we had brief descriptions of how CFIR constructs manifested in that case. In the same matrix, we documented considerations which we felt were important to project implementation but were not adequately captured using CFIR. All vignettes and matrices were developed through consensus by at least 2 individuals with deep case familiarity (PI, study coordinator, and/or student advisory board member) Fig. 2.

### Data analysis

The full team held 16 critical reflection meetings between January 2024 and May 2024. We used our vignettes and matrices to identify any CFIR constructs that were consistent across cases *and* which we felt were likely to be both unique and important to CSV research. We then identified consistent themes that were both important considerations for implementing CSVI work but were not surfaced by CFIR. We documented these semi-structured meetings with meeting minutes and reflexive memoing. We synthesized our vignettes, matrices, memos, and meeting notes to create a concept map and checklist tool. If, during the analysis process, the authorship team identified a knowledge gap related to a particular criteria or case, we reached out to research assistants and advisory board members from the original study team to identify an additional co-author who could provide the necessary information.

#### Intimacy and Sexual Health Intervention Pilot for University Students (ISHIPUS)

##### Study team included: RFM, AVV, VO

ISHIPUS was a randomized control phase 2 trial to assess the potential effectiveness of a sexual wellness and partner intimacy app [24] among undergraduate students at the University of California. The project took place in the 2023–2024 academic year. Two hundred and seventy-five students were enrolled in the study beginning in the fall of 2023 and randomized to the intervention or control arm. The intervention arm received access to the app for 1 month, with follow-up immediately following intervention completion and 1-month post, ending in May 2024. During the 1-month intervention period, participants completed brief weekly engagement surveys to assess how frequently they engaged with the app and which content they had engaged with that week. The study was powered to detect a difference in partner compassion and consent communication. The app is evidence-informed and was developed in consultation with academic experts in sexual health, sexuality education, and sexual trauma. It was originally developed as a commercial enterprise for women in their 30 s and 40 s and includes exercises and content to improve sexual health and wellbeing.

RFM made initial contact with the campus sexual assault resource center at the end of the 2023–2024 academic year, when the research application was first developed and submitted for grant funding. Resource center staff were generally positive but did not directly promote recruitment as the app was not closely aligned enough with their central program mission. The study team was in regular contact with resource center staff to ensure that the data we collected could still be internally useful and consistent with their mission to provide sexual assault advocacy and education for the campus community. Working with a doctoral student, RFM convened an undergraduate SAB in the beginning of the 2024–2025 academic year to support survey development and recruitment, and to provide overall input into the research question, data analysis, and intervention implementation. SAB members were subsequently invited to participate in data analysis and dissemination, resulting in several undergraduate research posters and ongoing manuscript development.

#### #GameChangers (#GC)

##### Study team included: FS, SKS, RFM

#GameChangers/Baguculi Betintfo (#GC) was based at UNESWA and is an adaptation of the Enhanced Assess Acknowledge Act (EAAA) program, which has been shown to reduce college women’s risk of sexual assault by 50% for up to 2 years [25, 26]. Work conducted by FS, SKS, and RFM found that 51% of female university students at UNESWA have experienced a sexual assault in their lifetimes, 38% experienced sexual assault in the previous 12 months, and 29% of women who reported lifetime SV also reported current symptoms of PTSD related to the assault [27].

The project included an adaptation and phase 1 pilot to assess potential effectiveness, feasibility, and acceptability. We recruited 201 female participants for a planned waitlist control effectiveness study; however, due to COVID-19 related disruptions, only one group of 19 participants completed the intervention. Additional details on EAAA and our pilot project are available elsewhere [7, 25–28].

#GC focused on SV resistance rather than prevention, using an explicitly feminist framework that acknowledges and rejects social-cultural aspects of victim-blaming rape culture [29]. The intervention adaptation and pilot were the second phase of a two-phase project funded by the Sexual Violence Research Initiative (SVRI) from 2017–2020. In early 2018, RFM, FS, and SM conducted a mixed-methods study on the prevalence and correlates of SV at the UNESWA [27]. That study included key informant interviews with campus administrators and stakeholders to understand campus context and relevant policies. These data were used to tailor #GC and to build relationships that laid the groundwork for #GC’s implementation and YMNN’s later emergence (see below). Intervention recruitment began in late 2019, immediately following dissemination of the CSV study, which likely increased interest. Although originally intended as a full randomized control trial, the project was truncated to a 1-group pre-post design due to the COVID-19 pandemic.

FS, SM, and RFM attended the Sexual Assault Resistance Education Centre training of trainers in Windsor, Canada, and then implemented a facilitator training at the University of Eswatini. They contracted with a local siSwati speaking counselor to provide psychological support for staff, facilitators, and study participants as needed. All facilitators and study participants were provided with a toll-free number they could use to reach the counselor at any time, with hours billed directly to the study team. FS also provided frequent informal debriefings with the facilitators and served as a backup source of mental health support.

#### Yilamu Mfundzi Ngci Ngami (YMNN)

##### Study team included: FS, SKS, MTT, AVV, VO, RFM

Following the UNESWA campus climate survey, university administrators asked FS and SKS to finalize the draft of a campus-wide sexual harassment policy [30]. In early 2021, FS, SKS, and RFM then collaborated on a research proposal to the Global College Campus Violence Prevention Program (GCVP) for an online, asynchronous curriculum to implement the mandatory training called for in the policy. In October 2021, after securing GCVP funding, the Principal Investigators (PIs) met with a student stakeholder group, the Student Network Against Sexual Harassment (SNASH). We learned that SNASH was already engaged in similar work and with the support of our funder, FS, SKS, and RFM chose to support the refinement and evaluation of the work already being done on campus rather than creating a new intervention.

Yilamu Mfundzi Ngci Ngami (“Help Student, It stops with me”) is a WhatsApp-based peer-led intervention that aims to reduce the acceptability of sexual harassment at UNESWA. It was developed and pilot tested for feasibility and acceptability with a small group of students (*n* = 9) recruited from all 3 of UNESWA’s campuses across Eswatini. At the beginning of the intervention development process students identified 3 core principles that would underpin all content: empathy, respect, and justice.

The intervention’s concept, format, and content were conceptualized mainly by students, led by MTT, with day-to-day support from AVV under the supervision of RFM, FS, and SK. The intervention was planned for a 10-day delivery period in which a new module was delivered over WhatsApp each weekday to a group of approximately 10–15 participants. However, we expanded the delivery period to 3 weeks to accommodate national protests and students’ exam periods. A trained peer facilitator moderated the group discussion and encouraged conversation through predesigned prompts. The 10 modules were: Let’s get to know one another; Let’s communicate; “Consent; Gender Based Violence Spectrum; Reporting; Imagine – in which participants were encouraged to imagine restorative and liberatory approaches to addressing campus GBV; Supporting a friend; Deconstructing Rape Myths, Gender/Sexual Minorities, and Commitment to Ending Gender Based Violence – a call to action and pledge to continue the conversation after intervention completion. Content development and theater testing took place in an overlapping, iterative manner to maximize project efficiency (see Supplementary material 2).

For the feasibility pilot, AVV and MTT trained intervention facilitators using a co-designed manual. Trainees were presented with the purpose of the intervention, their roles, module contents, and facilitator techniques. The study team provided daily airtime bundles to participants throughout intervention delivery. AVV regularly checked in with pilot facilitators to discuss challenges and strengths of the intervention. Feasibility and acceptability were assessed via debriefs with facilitators and a qualitative thematic analysis of the chat records.

#### Ntombi Vimbela! (NV!)

##### Study team included: MM, PM, YS

Ntombi Vimbela! (NV!) was a multi-campus South African intervention developed and implemented between 2018 and 2020. A survey conducted by the team among female students aged 18–30 from six Technical Vocational Education and Training (TVET) college campuses and from three university campuses found that 20% of participants experienced SV in the past year, with 17% reporting partner SV and 7.5% reporting non-partner rape. Individual-level factors that increased female students’ vulnerability to SV included being a first-year student, coming from a poor socio-economic background, lacking food and material resources, engaging in risky sexual behaviors such as having multiple partners and transactional sex, experiencing mental ill-health and harmful alcohol use, and having limited awareness of sexual assault risks—particularly in social settings with high alcohol or substance use. Additionally, acceptance of gender-inequitable and victim-blaming beliefs, reduced power in unequal sexual relationships, abuse of power and sexual entitlement by male staff, and female students’ emotional barriers further heightened vulnerability [8].

Following the survey and qualitative formative research, the team worked on intervention development and an initial pilot test involving 17 peer facilitators followed by a non-randomized pilot feasibility study across eight technical colleges and universities in five of South Africa’s nine provinces. Ninety-eight participants completed in-person baseline assessments and endline focus group discussions. Remote follow-up surveys and telephonic in-depth interviews were conducted by research assistants with 35 participants one-year post-baseline, coinciding with COVID-19 lockdown regulations. Additional details regarding NV!’s content, development, and outcomes are published elsewhere [8, 31]. The program focuses on sexuality empowerment, gender and social norm change, early identification of sexual assault risks, self-defense, resistance strategies, and mental well-being. The theoretical underpinning of NV! draws from formative research findings as well as evidence-based programs such as Stepping Stones Creating Futures [32], SASA! [33], and EAAA [25].

NV!’s content was tailored to address the unique challenges faced by South African female students in higher education and implemented over ten weeks, with one session delivered weekly in campus venues outside regular academic schedules. Refreshments were provided at each workshop. The PIs and project coordinator observed the delivery of workshops and convened weekly debriefing meetings to gather facilitator feedback on their experiences managing group dynamics and delivering content. Facilitators received ongoing support from the PIs and project coordinator to enhance their understanding of CSV and NV! content and potential vicarious trauma due to participant disclosures or being triggered into their past traumas. All project staff could access the employee wellness program to access counselling support. Participants were provided with lists of campus services and local violence support service providers.

## Results

In the first round of critical reflection meetings, we used inductive coding based on the CFIR tool to populate analytic matrices and identified issues that were highly salient across all case studies, paying special attention to those we felt either were not sufficiently captured through CFIR, or were likely unique to CSVI research (see Supplementary material 1: Table S1 for an abbreviated example). We identified 6 key domains that are unique to CSV IS. We situated these domains within a conceptual model to highlight how they interact within a broader social ecology (see Supplementary material 1: Fig. S1’ for worksheet with example notes based on case study vignettes). From this conceptual model we created a checklist tool for each domain. The checklist includes questions we encourage CSVI teams to ask themselves in the design and implementation phase of a study. For three domains – context, infrastructure, and planning – we specifically highlight questions study teams should assess in the planning stages as well as suggested documentation points to consider how certain issues may have impacted data collection and intervention delivery, with attention to their potential influence on study generalization and intervention transferability. We also provide a set of reflection questions to consider impacts across larger domains.

### Policy and legal framework

CSVI research can have unique policy and legal concerns that go beyond other standard biomedical or behavioral interventions. We found that the inner (institutional) and outer (socio-legal) contexts were often intertwined. Many universities mandate sexual harassment or violence trainings; however, none of the institutions where our case studies took place mandated that these interventions be evidence-based. Two of our interventions – NV! and YMNN – were developed for specific institutions. As such, the content, contact-hours, and format were aligned with government and university policies. The other two – ISHIPUS and #GC – were adapted from pre-existing evidence-based or informed programs. While the latter were more likely to have pre-tested internal validity (effectiveness), the lack of alignment with institution policy presented barriers to implementation at scale.

SV sits at the intersection of public health and criminal justice. As a result, stakeholders and study teams sometimes had differing definitions of SV, harassment, and assault. Institutional and legal definitions of SV or sexual harassment (SV/SH) may not align within a single study site, much less across institutions and countries. In our case studies, the variety of definitions presented challenges that ranged from the relatively minor – providing additional clarity in an academic manuscript – to major challenges disseminating study findings and generating long-term buy-in if stakeholders did not think that the intervention’s primary outcome was in alignment with campus priorities.

Mandated reporting was a frequent policy challenge in our case studies. SV disclosure policies vary significantly across contexts and jurisdictions, and some university employees and healthcare providers are required to report SV/SH disclosures to appropriate authorities, regardless of the survivor's age or wishes. These policies can create barriers to open communication and trust between survivors and study staff, potentially discouraging individuals from seeking help or participating in interventions. In the ISHIPUS project, if SAB members disclosed a personal experience of sexual assault or violence, paid study staff would have been obligated to report their experience to the appropriate office, regardless of whether the student wanted the incident to be reported. We were careful to brief SAB members on this rule at project initiation, with occasional reminders as necessary. While these reminders led to rich discussions on the nature of trauma-informed support for survivors, they may also have stifled any survivors on the SAB from freely discussing aspects of their lived experience that could have enriched the project. In Eswatini, any witnessed or suspected sexual offences must be reported to law enforcement regardless of the survivor’s age. However, it is unclear how the law applies in research contexts. This ambiguity required the research team to collaborate closely with ethics committees and institutional review boards to ensure compliance with local laws while adhering to trauma-informed best practices. Such collaboration is essential not only for legal compliance but also for maintaining trust with participants and ensuring that the intervention remains sensitive to the needs of survivors.

### Praxis

*Praxis* encompasses both the innovation (intervention) itself and the process of project implementation. Notably, while the CFIR tool focuses on the importance of culture in the institution that plans to implement an innovation, we found that it was equally important to reflexively consider the culture of the innovation and study team. All 4 case study interventions were grounded in feminist and trauma-informed principles. These principles are about power: Supporting survivor’s power and agency and deliberately naming the (imperialist white-supremacist capitalist) hetero-patriarchy [34] as the root cause driving SV/SH against women and gender minorities [22, 35, 36]. These principles in our *innovations* – and their power analyses – were also considered part of our project implementation *process*. Praxis requires study team members to consider if the way in which a study is implemented is consistent with the principles undergirding the intervention.

In 3 of our case studies, the research institution held more financial, social, and professional capital than the institution hosting the intervention. Two of these interventions (#GC and YMNN) were collaborations between the United States and Eswatini, and one was a collaboration between the South African Medical Research Council and regional institutions of higher learning (NV!). These inequitable power dynamics were not always acknowledged during project implementation. It is unclear to what extent these disparities may have affected equitable collaboration, resource sharing, accountability, and overall implementation success, nor is it clear how the uneven weighting team member expertise may have impacted study rigor or potential impact.

Distribution of leadership roles emerged as an important component of project praxis. In YMNN, the project PIs were unaware that SNASH, a student group at UNESWA, was already engaged in sexual harassment prevention work. When the PIs realized that SNASH had already developed an intervention similar to the one they had separately proposed, the PIs worked with their funder to rework their original proposal, partnering with and supporting the students to expand and evaluate their existing work. Students were offered paid leadership positions and integrated into study design and dissemination efforts.

We also noted the importance of proactively identifying perspectives that were missing from the project team. As YMNN moved forward, student leadership recognized that LGBTQ perspectives were missing. The team reached out to an off-campus advocacy group to incorporate their research and materials into the intervention. The content was well-received during the pre-test phase. However, no SAB members or pre-pilot participants identified as LGBTQ, which team members felt hindered their ability to determine if the content was truly acceptable and trauma-informed. As a result, this module was not included in the final set of materials. At the time, the team felt that this was the best choice to avoid inadvertently creating harm. However, the problem could have been avoided if project PIs had noted the lack of representation earlier in project design and implementation.

Explicitly naming values makes it easier to enact them. The NV! intervention and study team culture were developed to align with the cultural values of ubuntu and sisterhood. Facilitators were trained on feminist values and ethical research practice, and vernacular/home languages were embraced and used throughout project implementation. In #GC, leadership emphasized trauma-informed approaches for intervention, data collection, and workplace culture, providing debriefing opportunities and connections to formal psychological support for research participants and staff members alike.

One of the most measurable ways to assess project praxis is in the budget. Resource constraints often lead to low wages for entry level research staff, and international and/or intra-institutional red tape can lead to delayed payment processing. Taken together, this low and late pay symbolically and literally undervalues the contributions of team members with the least social power (young, less formal education, often female and based in the global South) while recreating the financial stressors that contribute to violence exposure for these same communities.

### Relationships

CFIR’s single inner setting construct did not capture the complexities of collaboration within and across multiple settings in CSVI work. Instead, we focused on CSVI research as an opportunity to build and deepen multi-site, multi-stakeholder relationships that can contribute to the long-term work of reducing SV at scale.

In all 4 cases, our teams were not the first or only entities working to implement CSVIs on the study site campus. As early as possible, research teams should identify who at the study site campus is already engaged in CSV work. Building relationships with these stakeholders has multiple benefits: in the planning stages, pre-existing projects have likely already collected important formative data, allowing the research team to build on what already exists, rather than duplicate existing efforts. Should the new intervention prove to be effective, pre-existing stakeholder buy-in will likely be key to long-term, sustainable implementation at the study site.

The NV! project was a new collaboration between research and study site institutions, and so the study team had to build new relationships with study site stakeholders from the ground up. Previous government commitments to addressing CSV increased site stakeholder willingness to collaborate on the project; for some, hosting NV! was useful because they could use it as evidence of their institutional commitment to address CSV. Similarly, YMNN was developed as a continuation of many years of collaborative work, with strong buy-in from the study site’s administrative leadership. Pre-existing trust between FS, SM, and student groups allowed for a rapid and smooth pivot from the original intervention to adaptation and pilot test of the student-created project.

### Context

Issues related to *individuals, outer setting*, and *process* often overlapped in our case studies. We treated these as a single overall construct, which we labelled *context*. Distinct facets of the research *context* were salient at different points in project planning and implementation.

All four projects in in all three countries had to navigate critical incidents such as student strikes, campus protests, and/or national debates on sexual harassment and violence. In South Africa, protests at the start of the academic year delayed NV!’s implementation, which led to logistical complications with campus calendars and space availability. These events also influenced the perceived value and urgency of the work.

External events may impact the likelihood of survivor disclosure during data collection and intervention delivery, with distinct effects for each setting. In Eswatini, the Sexual Offences and Domestic Violence (SODV) Act passed midway through data collection and we found that participants who completed the survey following SODV passage reported a significantly higher rate of SV than participants who completed the survey before its passage. In group intervention settings, survivor disclosures – and how these are handled by facilitators—may also impact group dynamics, meaningfully influencing the intervention’s delivery and quality. Documenting these events is crucial to accurately interpret later evaluation data.

Funder priorities shape a project’s focus and scope but are not always aligned with campus priorities. Study teams must navigate multiple stakeholder priorities while maintaining a commitment to rigorous scientific practice. In each of our case studies, study teams chose to collect data on additional outcomes beyond violence because they were perceived to be higher priority for funders. Two case studies were supported by funders whose primary aim was to address campus violence. The other two were funded with mechanisms that primarily focused on HIV and mental health. This necessitated careful planning to create interventions that met campus and funder goals while remaining consistent with the existing evidence base for CSVIs. The NV! project, for example, purposely developed an intervention and generated evidence that addressed the intersection of violence, substance use, mental health, and HIV. ISHIPUS collected data on compassion and mental health in addition to violence and consent.

In addition to generating pilot data for later funding proposals, CSVI research findings must also be compelling for local stakeholders to support ongoing partnerships and eventual scale-up. In each case study, the research team was careful to consult with different stakeholders to understand what data and findings they might find most compelling. What do students, administrators, fellow SV/SH interventionists working at or near the study site institution need to know to be assured a project is salient and valuable? In addition to securing ongoing collaborations, early consulting with campus stakeholders provided an opportunity to support ongoing programmatic work, building relationship and rapport. We found that relatively small additions to a study protocol – for example, including a survey item related to a program’s acceptability, or the impact of a campus-wide program tangentially related to CSVI – could result in a large value-add for local groups that do not always have the technical training or research infrastructure necessary to generate these data alone. The type of data that stakeholders will find most compelling and useful will vary widely across projects. We found that the best way to know what stakeholders wanted was to ask them, either formally (key informant interviews or focus groups), or informally by simply being in relationship.

### Infrastructure

CSVI research has specific physical, technical, and psycho-social infrastructure requirements for rigorous and ethical implementation.

Two projects incorporated physical self-defense training (NV! and #GC). These required campus venues that were convenient, safe, private, comfortable, and big enough to accommodate the group. Teams also had to secure campus offices for study administration, data collection, and storage space for intervention materials. Since the host institutions already struggled with space constraints, it was important to identify our specific needs early, ensure host institutions appreciated the needs, and then negotiate carefully to ensure minimum disruption to day-to-day campus activities Table 1.

**Table 1:** Campus sexual violence intervention research implementation checklist

Policy and legal framework
Are there pre-existing policies at the host institution regarding the type, nature, and frequency of sexual violence or harassment interventions?
*Operationalization of SV/SH*
How is SV/SH defined by the institution? Regional law? National Law?
How is SV/SH defined by the proposed intervention?
How is SV/SH operationalized and measured within the study protocol?
Are the legal, intervention, and research definitions of SV/SH aligned? If not, which definition will take precedence? Will this vary by context?
*Mandated reporting*
Are there mandatory reporting policies? Can they be offset and if so under what circumstances?
Who is ultimately responsible for ensuring compliance with mandated reporting policies?
Praxis
What social identities are represented on the leadership team? What are the power dynamics?
Is project leadership meaningfully accountable to the population the project seeks to serve?
Who was involved in writing the grant? Designing the study? Selecting the outcome measure?
What are the team’s values and ideal work culture? How will this be promoted?
Are staff being paid a livable wage in a timely way?
Do all team members have the opportunity for meaningful professional development?
Relationships
What relationships exist between the research institution and study site? What institutional power dynamics might be at play?
Who is already doing similar work on the campus in the community? Is the study team in relationship with these stakeholders? Has there been a meaningful effort to build relationship?
How will the proposed project engage with pre-existing expertise and efforts to identify synergies and avoid duplication?
What are the current relationships between students, administration, faculty, staff, and other stakeholders at the study site? How might this impact the proposed work?
Context
* Planning*
Is it foreseeable that one or more critical incident (protests, strikes, legal changes, national discourse) might occur during the study timeline? Is there a plan for how some of these might be mitigated?
Is sexual violence the (proposed) funder’s primary focal area, or are there multiple project outcomes?
Is there an opportunity to collect data that could support pre-existing efforts to address SV/SH?
Who are key stakeholders? What kinds of evidence do they find most compelling?
Implementation*
Did an SV/SH critical incident take place on campus or in the broader community during study activities?
Was there a disclosure that triggered a mandated report? Did the survivor want the incident reported?
Infrastructure
* Planning*
Does the physical intervention space provide sufficient privacy, space, and comfort?
Is the data collection and/or intervention delivery platform secure? How will participant privacy and safety be supported if data is collected or intervention delivered remotely?
What is the current standard of care and capacity for providing psychosocial support? Does the study team need to develop additional mental health infrastructure?
What psychosocial support is available for study team members?
What is the protocol for identifying and supporting participant emotional distress? Study team member emotional distress?
Implementation*
Did physical and technological infrastructure sufficiently support participant privacy?
Was the psychosocial support infrastructure utilized? If so, by who, and was it sufficient?
Were there major barriers or facilitators to psychosocial support uptake? Did these vary for participants and study staff?
People
* Planning*
Do all members of the study team understand and share the project’s axiological and theoretical framework?
Does staff training address affective learning goals?
Is the project sufficiently staffed to allow for cross-training and rest?
What professional development opportunities are available via the project, and to who?
Implementation*
Were the facilitators able to deliver the intervention in a way that was consistent with project axiology and theoretical grounding?
Were there issues with staff burnout or emotional distress? How were they addressed?
Did team members have sufficient role clarity? Were there clear boundaries between research, intervention delivery, and therapeutic roles? Did team members who took on multiple roles have the necessary support (professional and psychosocial) to do so?
Reflection
* Policy and legal framework*
How do study findings fit within current policy and legal concerns? Do study findings need to be shared with policy makers? What is the best way to reach them?
What kind of evidence is most likely to resonate with key stakeholders?
* Praxis*
Were team values implemented consistently throughout the life of the project? What were strengths in the project’s praxis? What can be improved in the future?
* Relationships*
How did the project create, strengthen, or damage relationships?
Who is best positioned to lead evidence dissemination? For which stakeholder constituency?
* Critical incidents (if any)*
Could the incident(s) have been foreseen? How did they impact the project? Is there a way to plan or adapt for potential similar future events?
* Implementation science*
Which aspects of the project were key to successful intervention implementation? How can they be measured in future implementation or hybrid designs?
How does the intervention need to be modified to improve sustainability? Is this specific to the research site or should the modification be included for all future project iterations?

^*^For all implementation aspects, document how consideration impacted data collection and intervention delivery. How does this influence study generalizability and intervention transferability?

Technical infrastructure needs differed across projects. NV! and #GC participants completed surveys in-person in a space that was private with research staff trained in emotional first aid available to provide support as necessary. YMNN and ISHIPUS used remote data collection (surveys via SMS or email), so surveys did not include items related to intimate partner violence or that could trigger emotional distress. For the YMNN WhatsApp based intervention, the curriculum development team had to be mindful of potential risks participants might face if they participated in group conversations related to SV/SH on their own phone, where we could guarantee neither anonymity nor confidentiality.

CSVI research requires a high standard of psycho-social care for participants and staff. This care infrastructure may not already exist in the host institution. If this is the case, teams may have to generate their own. NV! leveraged relationships with stakeholders to establish referral pathways for psychosocial and violence support programs for study participants. The study protocol also included collective care structures for facilitators that included a workplace mental wellness program and weekly virtual group debriefings facilitated by PIs. In Eswatini, the #GC team contracted with a professional counselor in private practice to ensure adequate psychosocial support for all participants and staff. YMNN, while less emotionally intensive, provided emotional first aid training for intervention facilitators and regular self-care debriefings.

### People

One of the earliest emergent themes was the vital importance of cultivating a team that shares similar values and theoretical understandings regarding the roots of GBV.

Facilitators are perhaps the single most important determinant of CSVI effectiveness. Facilitators must not only refrain from victim-blaming or reinforcing harmful gender stereotypes, but they must also be able to actively identify and push back on these concepts when they arise in group discussions. Moreover, they must be able to do so with emotional and cultural sensitivity to ensure that participants continue to feel welcome and engaged in the intervention programming. YMNN, NV!, and #GC all required facilitators who were willing and able to engage in critical personal and social reflection on the root causes of GBV. Trainings included affective and cognitive learning outcomes so that facilitators (and all study team members) could reflect on and address internalized harmful gender norms before engaging with intervention participants.

CSVI work is emotionally intensive and staff have a high risk of burnout [37]. In resource constrained environments, staff may have to frequently shift roles between facilitator, researcher, and therapeutic provider. Sufficiently staffing the project and utilizing cross-training increases the likelihood that team members will be able to utilize time off when they most need it, reducing burnout and likely improving data quality. Cross-training and professional development opportunities for all team members also provides study teams an additional opportunity to consider their overall praxis through capacity building and professional development.

### Implementation and reflection

After reviewing our four case studies, we identified unique aspects of intervention implementation that can have a meaningful impact on data quality or intervention delivery and vary significantly from setting to setting. We also identified post-research tasks that can build relationships, increase study impact, and ensure that the project’s praxis remains consistent with the intervention’s theoretical underpinnings. Tracking implementation issues was useful at all stages of CSVI development, pilot testing, and evaluation, not just in the final stages of effectiveness testing.

It is essentially impossible to run a ‘true’ efficacy trial for CSVI interventions. The complexity of a college campus social ecosystem cannot be simulated in a laboratory or computer model. For that reason, we argue that all CSVI research is implementation research to a greater or lesser degree.

## Discussion

We conducted a comparative case study of four CSVIs and analyzed these using a CFIR lens to develop an IS checklist and planning worksheet for CSVI research. The checklist and frameworks are intended to serve as tools to guide researchers, program developers, funders, policy makers, and other stakeholders to consider contextual elements that are likely to vary across CSVI settings and substantially impact the effectiveness of CSVI implementation and/or evaluation. While the four case studies we selected are not exhaustive, the rich contextual descriptions we developed based on our teams deep familiarity with each case and our use of the CFIR framework to bound our cases and initial inductive coding significantly enhances the transferability of our findings [21]. We view the conceptual framework and checklist as generative rather than exclusive – they are designed to guide planners and practitioners navigating the ways in which the topic of SV can impact a study’s planning and implementation. For example, institutional review boards may choose to use the tool as a lens to identify potential risks or adverse events unique to CSVI research. Funders may find it a useful framework to consider how to allocate scarce resources or to evaluate potential impact among multiple highly competitive applications.

Our case study methodology sought to expand the boundaries of IS for CSVI both as the intervention and as part of its broader context of study/intervention implementation. We sought to provide CSVI researchers a framework and tool that can be used in hybrid implementation/effectiveness study designs, addressing implementation concerns for these complex interventions sooner rather than later. In an environment of constrained resources, in which every research dollar and hour of participant time must be used as efficiently and respectfully as possible, we argue that there is a pressing imperative to systematically apply robust IS frameworks in future work, ensuring that violence prevention interventions are not only feasible and acceptable but also successfully integrated, scaled, and sustained in campus settings.

Despite the progress made in applying IS frameworks to CSVIs, several gaps remain that warrant further exploration. Future work should focus on refining methodologies for capturing the impact of legislative changes on ongoing programs and data collection efforts. Additionally, there is a need for strategies to effectively capture implementation data that reflects sustainability, equity, and power-sharing among stakeholders throughout the design, implementation, and community engagement processes. Ensuring that CSVIs are truly inclusive, trauma-informed, and impactful in diverse contexts is paramount. Addressing these areas will enhance the effectiveness of CSVIs and contribute to broader violence prevention efforts.

The application of IS frameworks is essential for enhancing the effectiveness of CSVIs. These frameworks provide structured approaches that facilitate the design, execution, and evaluation of hybrid projects, ensuring that interventions are not only evidence-based but also contextually relevant. By integrating IS principles, researchers can better navigate the complexities of campus environments and address the multifaceted nature of SV. We anticipate that our checklist and worksheet can support the rigor of CSVI research by helping to identify potential threats to external validity early in the study design process. The high degree of contextual variability in CSVI research necessitates study designs that can simultaneously account for internal and external validity, rather than the more traditional biomedical model of assessing these in a systematic, sequential order [13].

Hybrid designs that can account for both simultaneously are likely to be an important methodological step forward in the field. Curran et al. suggest that researchers consider four questions when deciding how to best balance effectiveness and implementation research questions: current effectiveness evidence-base for the chosen intervention, expected need for adaptation across sites, how much is known about the site where the intervention will be implemented, and the degree to which the research team feels prepared to assess different implementation strategies [5]. These questions are aligned with the themes we identified in our case studies. For example, even if an intervention is already widely in use – as is the case for institutions that have pre-existing CSVI mandates – researchers should first stop to consider if there is pre-existing evidence of effectiveness for the intervention, and, if so, how effectiveness has been defined and according to which metrics. At the same time, while it is important to have a clearly defined outcome measure, the wide variety of SV/SH operationalizations across institutions, jurisdictions, and fields suggests that researchers must be mindful to incorporate multiple measures of “success” that can speak to multiple stakeholder audiences. This aligns with recent expert stakeholder guidance on the evaluation and reporting of complex interventions, which emphasized that, “researchers should answer the questions that are most useful to decision makers rather than those that can be answered with greatest certainty.” [13].

The model and checklist we developed demonstrate significant utility in guiding CSV projects across various contexts. As illustrated by our case studies, these tools facilitate reflection on critical implementation factors, enabling research teams to adapt their strategies effectively. For instance, by incorporating qualitative feedback mechanisms into data collection, projects enhanced community relationships and improved sustainability. The model also emphasizes the importance of aligning intervention objectives with broader health issues, such as HIV and mental health, which can attract additional funding opportunities. Overall, the checklist serves as a practical resource to ensure that interventions remain responsive to stakeholder needs while adhering to best practices in IS.

The questions of power, praxis, and relationship arose frequently in our case study analyses and infuse much of the checklist and planning tool. Global public health has only recently begun to grapple with questions of epistemic violence, critically examining the ways in which ongoing legacies of colonialism impact every aspect of the field from where funding is allocated to what types of data count as meaningful evidence [38]. SV researchers in the global South have been at the forefront of this movement, emphasizing the importance of anti-colonial and intersectional feminist values and praxis [39, 40]. Our case studies suggest that continuing to name, document, and critically reflect on how issues of power, relationship, and praxis are not only ethical imperatives, but will meaningfully improve the quality of our science.

## Conclusions

Leveraging IS frameworks like CFIR is vital for advancing the field of CSVIs. By developing tailored models and checklists, researchers can navigate complex environments more effectively while remaining aligned with contextual priorities. Continued engagement with stakeholders and ongoing reflection on gaps and opportunities within the proposed model and checklist will be essential for ensuring that CSVIs evolve to be impactful, scalable, and sustainable in diverse campus environments.

## Supplementary Information

Supplementary material 1: Table S1: Sample notes from inductive analytic matrix. Figure S1: Conceptual framework and planning worksheet with notes from case study analysis.

Supplementary material 2: Yilamu Mfundzi Ngci Ngami! Facilitator Handbook.

## Data Availability

All data supporting the findings of this study are available within the paper and its Supplementary Information.

## Abbreviations

CFIR: Consolidated Framework for Implementation Research
CSV: Campus Sexual Violence
CSVI: Campus Sexual Violence Intervention
EAAA: Enhanced Assess Acknowledge Act
EBIs: Evidence-based interventions
GBV: Gender-based violence
#GC: #GameChangers
IS: Implementation Science
ISHIPUS: Intimacy and Sexual Health Intervention Pilot for University Students
LMIC: Low- and middle-income countries
NV!: Ntombi Vimbela!
PI: Principal investigator
SAB: Student Advisory Board
SNASH: Student Network Against Sexual Harassment
SV: Sexual violence
SV/SH: Sexual Violence/Sexual Harassment
SODV: Sexual Offences and Domestic Violence Act
UNESWA: University of Eswatini
YMNN: Yilamu Mfundzi Ngci Nami
