# Publisher Correction: Case study guided development of an implementation science framework and checklist for campus sexual violence intervention

**DOI:** 10.1186/s44263-025-00232-z

**Published:** 2026-02-05

**Authors:** Rebecca Fielding-Miller, Anh Vo, Vinton Omaleki, Pinky Mahlangu, Yandisa Sikweyiya, Fortunate Shabalala, Sakhile Masuku, Menelisi T. T. Dlamini, Mercilene Machisa

**Affiliations:** 1 Herbert Wertheim School of Public Health and Human Longevity Science, University of California, San Diego, USA; 2 Division of Infectious Diseases and Global Public Health, School of Medicine, University of California, San Diego, USA; 3 South African Medical Research Council, Cape Town, South Africa; 4 Department of Community Health Nursing, University of Eswatini, Kwaluseni, Eswatini

**Publisher Correction: BMC Glob. Public Health 3, 106 (2025)**

**https://doi.org/10.1186/s44263-025-00215-0**

Following publication of the original article it was reported that the ‘Competing Interests’ declaration needed to be updated.

The previous Competing Interest statement was: The authors declare no competing interests.

The updated Competing Interest statement is: MM is a guest editor for the collection ‘Advances in sexual and gender-based violence prevention at institutions of higher education’ and an Editorial Board Member of BMC Global and Public Health.

The original article has been corrected.

