# Supplementary material for: Case study guided development of an implementation science framework and checklist for campus sexual violence intervention
Source: BMC Glob Public Health. 2025 Nov 25;3:106. doi: 10.1186/s44263-025-00215-0 (PMC12649002; doi:10.1186/s44263-025-00215-0)

**Table S1: Sample notes from inductive analytic matrix**

| **Outer setting** | | | | | |
| --- | --- | --- | --- | --- | --- |
|  | *YMNN* | *#GC* | *ISHIPUS* | *Ntombi Vimbela* | *Unique concerns?* |
| Critical incidents | Political unrest  Student protests/strikes | SODV bill passed immediately prior to pilot  COVID-19 pandemic truncated RCT to pilot  Student activism and strikes related to alleged misconduct of university staff member | Student encampments in Fall 2023, political unrest  Ongoing graduate student strike activity throughout Spring 2024 | Student protests and strikes  Conflicts with sporting events required scheduling flexibility | Student activism  Impact of outside sociopolitical events on project salience |
| Policies and laws | The initial conceptualization of the intervention was centered around UNESWA Sexual Harassment Policy.  Delays from the local IRB review led to fielding study as programmatic pilot rather than initially planned RCT. | Mandatory reporting laws were unclear which made it hard to know what to do programmatically and for IRB review | State mandates that universities provide consent education, but does not require the education be evidence-informed  All UC employees are mandated reporters with exceptions for research. Exceptions do not apply to conversations with student advisory board | Revised Amended Sexual Offences Act states that women under the age of 25 in higher education or living in a res are defined as ‘vulnerable’ and places an obligation on anyone how has ‘knowledge, reasonable belief or suspicion’ that a person has been a victim of a sexual offence to report this to the police. | Mandatory reporting  Difficulties aligning policy requirements between IRB, local legal requirements, and ethical trauma-informed research practices.  Significant – sometimes contradictory – variety in defining sexual violence across different socio-political environments |
| Social pressure | SNASH had already done the work of creating authentic demand, so we revised our plans to support and evaluate their intervention rather than creating something new. | Administration welcomed and supported project  Ongoing national conversation regarding sexual violence | SAB considered how student advocacy could be a source of pressure for long-term implementation | Following several incidences of GBV and femicide, student advocacy pushed to prioritize GBV on campuses, | Student advocates can be powerful allies  Important to assess and be in alignment with existing programming |

| **Inner setting** | | | | | |
| --- | --- | --- | --- | --- | --- |
|  | *YMNN* | *#GC* | *ISHIPUS* | *Ntombi Vimbela* | *Unique concerns?* |
| Culture | Team spent significant time developing core values that would ground intervention and study activities: Respect, Justice, and Empathy. | Intentionally cultivated research team culture to align with trauma-inform principals and centered protecting the wellbeing of staff and participants | Culture of research group (feminist), culture of implementation team (SAB), culture of department that allows team to do things, Sexual Assault Resource Center, culture of university (liability), Sanford funder culture | Built on local culture values of ubuntu, sisterhood, embracing diversity including allowing the use of home vernacular languages in implementation.  Facilitators were trained on feminist values and ethical research practice. | Numerous overlapping ‘cultures’ – intervention culture, study team culture, differing cultures in global North/South collaborations  Culture built by members of teams that allows them to interact with participants |
| Funding | Limited funding source but adequate to implement a small pilot. | SVRI funding was sufficient for pilot. Outlook for long-term funding was unclear | Very limited. Had to remove qualitative component to reduce labor costs. PI effort provided in-kind. | Partially funded through social impact bond. Supplemental funding from philanthropy. Implementing partner had in-scope operational funds. | GBV research is often low priority, creating need for multiple funding streams. Government funding supports larger projects. Primary costs are often labor. Actual effort typically exceeds budgeted effort |
| **Innovation** | | | | | |
|  | *YMNN* | *#GC* | *ISHIPUS* | *Ntombi Vimbela* | *Unique concerns?* |
| Source | Develop by students for students at UNESWA in collaboration with UCSD | Started from World Bank funding. Adapted innovation created by researchers in Canada | For-profit app with pilot funding from home institution. Access to app provided in-kind by app developers. | Informed by formative research with students on campuses, and adaptation of EBIs. | Multiple voices (facilitators and students) included |
| Evidence base | Theater testing in-person showed acceptability of project implementation in-person rather than online- however we haven’t pilot an in-person intervention yet | RCT demonstrated efficacy and effectiveness in North American contexts | Evidence informed, but not evidence based (need clarification). Worked with researchers to design but this app has not been evaluated. | NV! was piloted through a one arm pilot with the intervention group across 8 campuses in 5 provinces in SA. Students found the content of NV! relevant, appropriate for their setting. They reported that NV! empowered them with skills to assess and deal with sexual assault risky situations. | Important to clarify how we define evidence-based and what kind of evidence is meaningful to different stakeholders. |

| **Process** | | | | | |
| --- | --- | --- | --- | --- | --- |
|  | *YMNN* | *#GC* | *ISHIPUS* | *Ntombi Vimbela* | *Unique concerns?* |
| Teaming | UNESWA, UCSD, and SNASH worked on this project together. UNESWA faculty provided expertise on the policy, SNASH contributed on the context, facilitations and big picture ideas for the intervention and UCSD generate the contents and coordinate the pilot | Fortunate and Becca already know each other but haven't work together before. Wrote grants together. Fortunate connects Becca w/ everybody. Graduate students/peer navigators made up the SAB team | ISHIPUS utilizes a student advisory board that both advises the project process and SAB members work together in recruitment, problem-solving, testing. SAB is dividing up into smaller teams for data analysis | Team of implementors are students. The team of implementors are coordinated and guided by the researchers and PIs of the project. High involvement from research team by checking on all the sites. *Research team had to make sure facilitators understood the content through training and conducting debreifing meetings during implementation | How do we decide who to involve in the research project? How do we weight lived vs. trained experience? Do facilitators – and all team members – understand and embrace theoretical/cultural underpinnings of the project?  Teaming is a process- are stakeholders part of a team? Advisory groups? |
| Assessing Needs | Reassessed study design after engaging with student organization and they provided their needs. For instance, the intervention was initital do not have a prominant discussion component to it, after learning about SNASH's work in facilitating conversation through WhatsApp, we adapted this approach. | In 2017, Campus survey to understand prevalence and KIIs on campus | indepth interviews planned will ask participants about app use | Mixed methods formative research (survey and FGDs) with students on scope of GBV and drivers and context for how/why it happens. | Formative research. Assessing the needs to integrate needs into the intervention. What innovation already exist? But not many are evidence based. Do we need to assess what's already been done and what social movements are doing? goes back to epistemology of what counts as evidence. |

**Figure S1: Conceptual framework and planning worksheet with notes from case study analysis**


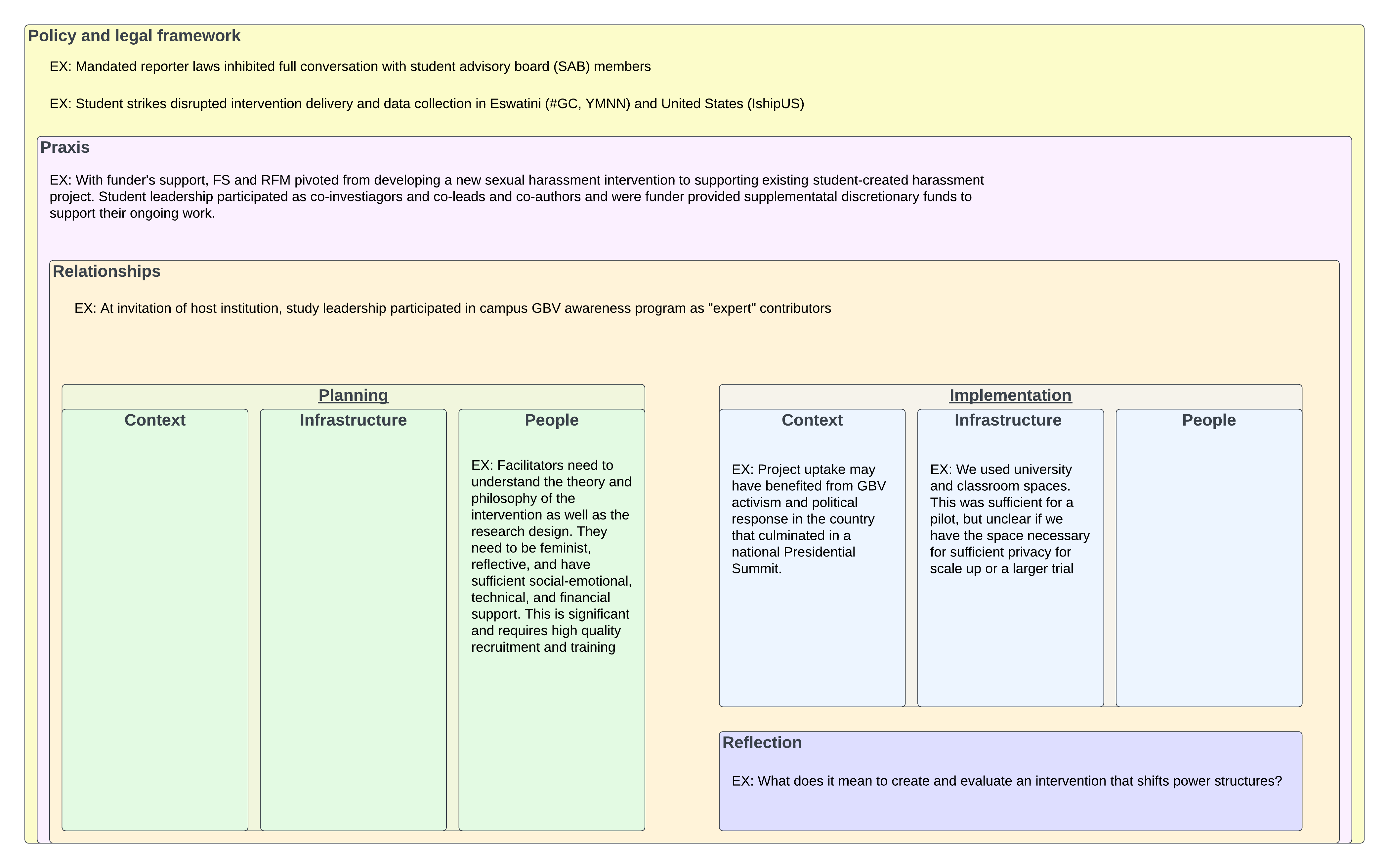

Supplement: Supplementary file 1 — Supplementary material 1: Table S1: Sample notes from inductive analytic matrix. Figure S1: Conceptual framework and planning worksheet with notes from case study analysis. [file 44263_2025_215_MOESM1_ESM.docx]
