# Supplementary material for: Case study guided development of an implementation science framework and checklist for campus sexual violence intervention
Source: BMC Glob Public Health. 2025 Nov 25;3:106. doi: 10.1186/s44263-025-00215-0 (PMC12649002; doi:10.1186/s44263-025-00215-0)
Supplement: Supplementary file 2 — Supplementary material 2: Yilamu Mfundzi Ngci Ngami! Facilitator Handbook. [file 44263_2025_215_MOESM2_ESM.pdf]

YILAMU MFUNDZI NGCI NGAMI! Facilitator Handbook

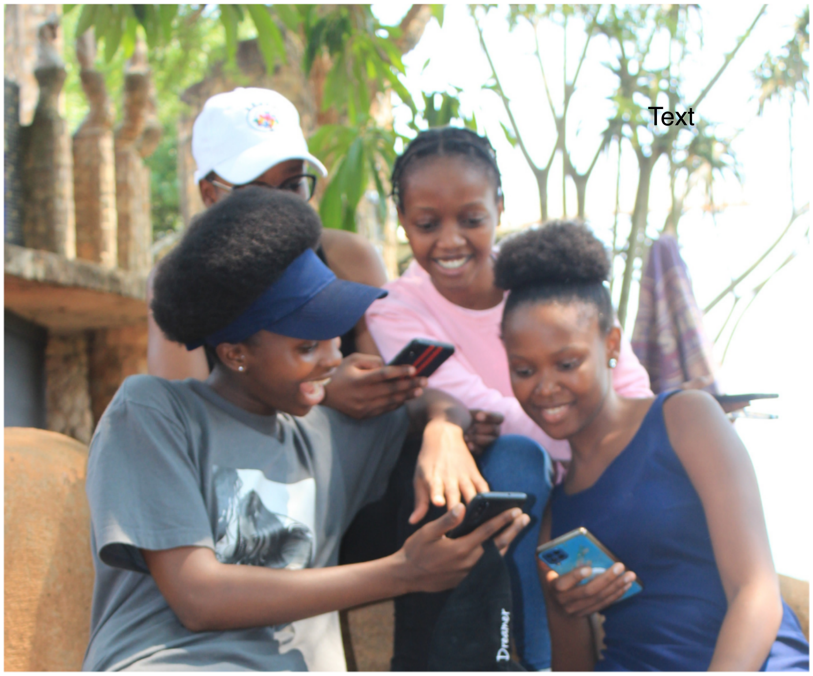

WRITTEN BY

University of Eswatini
University of California San Diego

Yilamu Mfundzi Ngci Nami: A sexual harassment prevention intervention for University students in Eswatini © 2021 by Fortunate Shabalala, Anh Vo, Rebecca Fielding-Miller, Sakhile Masuku, Menelisi T.T. Dlamini
is licensed under Creative Commons Attribution-NonCommercial-NoDerivatives 4.0 International.
To view a copy of this license, visit https://creativecommons.org/licenses/by-nc-nd/4.0/

Table of Contents

Table of Contents 2

Overview 3

Facilitator's Roles 3

Timeline 4

Structure of the Programme 4

Compensations: 5

Tips for Group Facilitation 5

Module 1: Let's get to know each other 7

Module 2: Let's Communicate 11

Module 3: Consent 12

Module 4: Gender Based Violence Spectrum 13

Module 5: Reporting 14

Module 6: Imagine 15

Module 7: Supporting a friend 16

Module 8: Deconstructing Rape Myths 17

Module 9: Gender/Sexual Minorities 18

Module 10: Commitment to Ending Gender Based Violence 19

Yilamu Mfundzi Ngci Nami: A sexual harassment prevention intervention for University students in Eswatini © 2021 by Fortunate Shabalala, Anh Vo, Rebecca Fielding-Miller, Sakhile Masuku, Menelisi T.T. Dlamini
is licensed under Creative Commons Attribution-NonCommercial-NoDerivatives 4.0 International.
To view a copy of this license, visit https://creativecommons.org/licenses/by-nc-nd/4.0/

Overview

The training manual outlines the Yilamu Mfundzi Ngcii Ngami programme, an intervention that is designed for students at UNESWA, that aim to:

1) Decrease the acceptability of sexual harassment
2) Gain awareness about UNESWA polices and resources related to sexual violence
3) Endorse fewer victim blaming rape myths
4) Have increased levels of self-efficacy to appropriately respond to disclosures of sexual violence experiences

This programme focuses on communication in relationships, power structure that shaped harassment, HIV testings, harassment policy, LGBTQ safety, rape myths and supporting people who has experienced violence. The programme aims to affirm existing knowledge of learners through dialogue. Because the intervention relied on participants' knowledge and experiences, the intervention is designed to spark conversations about ways in which students can end harassment on campuses.

Facilitator's Roles

As a Co-Investigator

The intervention is novel and we want to be able to adapt and alter the intervention as we go to ensure its effectiveness. If there is any issue that arises or any suggestions you like to make to improve the intervention; do not hestitate to bring them up.

- Provide suggestions and feedback to improve the intervention
- Identify issues and troubleshoot as needed
- Distributing and promote the surveys

As a Moderator

- Facilitators are expected to moderate the conversations on WhatsApp through [Insert date] to [Insert date]
- Each day, facilitators are expected to post the content for the day at 8AM and moderate the conversation throughout the day until 8PM.
- Probings and asking questions to reach the learning objectives using the Facilitator Handbook as a guide.
- At the end of each discussion (after 8PM), facilitators are responsible for compiling a short summary (either a paragraph or a voice note) of the conversation that took place and sharing the summary in the facilitator chat. The purpose of the short summary is that Vinton and Tina can synthesize the conversation across all the chats to be shared back with the participants the following day.
    - 3 most interesting things that you notices

Yilamu Mfundzi Ngci Nami: A sexual harassment prevention intervention for University students in Eswatini © 2021 by Fortunate Shabalala, Anh Vo, Rebecca Fielding-Miller, Sakhile Masuku, Menelisi T.T. Dlamini
is licensed under Creative Commons Attribution-NonCommercial-NoDerivatives 4.0 International.
To view a copy of this license, visit https://creativecommons.org/licenses/by-nc-nd/4.0/

- We will have a Facilitator Group Chat to support facilitators throughout the discussion. We encourage facilitators to help each other out if there is any question or when novel situations arise.
- Attend facilitator trainings: one 8 hours training session.

Timeline

Recruitments

Baseline Survey data collection

Randomization/ Finalise Roster

Intervention

Wrap Up Event

Immediate Follow up

3- month follow up

Structure of the Programme

- Groups: Each group will consist of 10 to 15 participants to one facilitator. The group will be led by students for students.
- Where: Anywhere with adequate internet, as the intervention will take place over WhatsApp
- Time of Day: The discussion will take place from 8AM to 8AM the following day moderated by the facilitators asynchronous throughout the day.
- For each module, the facilitators will post the contents assigned for the specific modules at 8AM.
- At the end of each day, the facilitators are responsible for compiling a summary of the discussion at 11:30PM and sending them to the Facilitator group chat for the US-based team to synthesize the content across all group chats.
- Before 8AM the next day, the US based team will share the synthesized summary with the facilitator to share with participants in their group chats before posting the content module for the following day.

Yilamu Mfundzi Ngci Nami: A sexual harassment prevention intervention for University students in Eswatini © 2021 by Fortunate Shabalala, Anh Vo, Rebecca Fielding-Miller, Sakhile Masuku, Menelisi T.T. Dlamini
is licensed under Creative Commons Attribution-NonCommercial-NoDerivatives 4.0 International.
To view a copy of this license, visit https://creativecommons.org/licenses/by-nc-nd/4.0/

Compensations:

Facilitators

- A stipend of E2500 will be disbursed to facilitators after all 10 modules are facilitated.

Participant

- Lunch Voucher
- Data vouchers

Guidance for Facilitators

Honor Diversity of identities, experiences and ways of knowing. We all have difference experiences and way in which we understand and experience the world. Connecting with each other may require stretching our minds and hears. Try listening with curiosity. Notice your own response to difference and discomfort.

Inclusive Language Invitation to share and use pronouns in English and use inclusive language that allows space for transgender, nonbinary and gender diverse people. This may be new to you and that's ok! The discussion is a great place to practice.

Asking someone's pronouns is a way of showing respect and avoiding incorrect assumptions about their identity. If it feels authentic when introducing yourself, consider sharing your pronouns and making your gender identity and other relevant aspect of your positionality explicit.

If you make a mistake, give a brief apology, correct the satement and carry on.

If you experience overwhelm During discussion about sexual harassment, it is common to feel reactions in our bodies, including physical sensations , emotions, needs, thoughts, relational challenges, etc. Refer to Emotional First Aid to distinguish if you need help or how you can navigate the experience.

When facilitating Do not include potentially traumatic graphic images as such images may act as a trigger for participants. Do not share others' stories and statements without asking for their permission. This includes victims' and survivors stories shared in the WhatsApp group.

Tips for Group Facilitation

Group dialogue is a cornerstone of this intervention therefore the facilitator play an important role. In our training, we discussed these principles and how we could use them to navigate each of the module and situations within each group chat.

Yilamu Mfundzi Ngci Nami: A sexual harassment prevention intervention for University students in Eswatini © 2021 by Fortunate Shabalala, Anh Vo, Rebecca Fielding-Miller, Sakhile Masuku, Menelisi T.T. Dlamini
is licensed under Creative Commons Attribution-NonCommercial-NoDerivatives 4.0 International.
To view a copy of this license, visit https://creativecommons.org/licenses/by-nc-nd/4.0/

It is important to note that everyone learns differently, some learn by talking about their thoughts while others take time to process what others say. Though it is important to encourage people who havent talk much in the group to share their thoughts, don't force them.

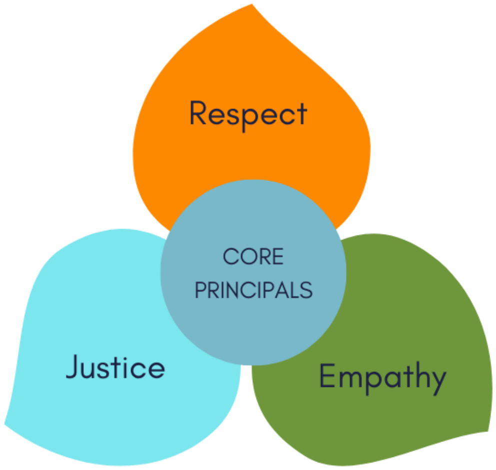

Being in an online environment can be challenging in that you cannot read their body language to gaze their engagement. As we continue to develop this intervention together through theater testing and piloting, we will become more aware of possible scenarios that arises and we will update this manual with your experiences and suggestions. Please use these suggestions are a resources and do no limit your creativity and your ability to use your experience to add to these suggestion.

The Whatsapp group is also a space for people to practice the skills we are discussing. I.e., this is where we can practice respectful, empathetic communication. Moderators need to be especially aware of comments that fall somewhere within our 'pyramid' and be able to call people 'in' rather than 'out' when these comments inevitably happen. This is a space for people to practice, not for them to be perfect.

Back to our core principals: respect, empathy, equity. Set ground rules on the first day and bring people back to them.

Probing/asking for clarification - the prompts are just the beginning

Be evidence based - if you don't know the answer, say so. Default to citations if you can.

Create a welcoming environment. - ask small questions, use people's names, make sure everyone feels engaged

Yilamu Mfundzi Ngci Nami: A sexual harassment prevention intervention for University students in Eswatini © 2021 by Fortunate Shabalala, Anh Vo, Rebecca Fielding-Miller, Sakhile Masuku, Menelisi T.T. Dlamini
is licensed under Creative Commons Attribution-NonCommercial-NoDerivatives 4.0 International.
To view a copy of this license, visit https://creativecommons.org/licenses/by-nc-nd/4.0/

Module 1: Let's get to know each other

Objective:
- To orient participants with the Yilamu Mfundzi course contents and the main learning objectives for each module.
- Create a welcoming environment that enable productive discussion about gender-based violence by building trust and familiarity among the participants and facilitator

Download Module Content

Instruction:
1. First, share the course summary and a spiel explaining the course

---

Example spiel:

Hello everyone, my name is ____. Thank you for your interest in participating in this training. Today, our main goal is to go over the course overview, getting to know each other and putting together a shared agreement (or "ground rule") on how we can approach this space.

Here is our course overview:
1. The Course summary are the learning objective over our next 16 days together (November 25-December 10), we will be discussion topics related to sexual harassment.
2. Each day, I still start introduce the topic of discussion at 8AM
3. This is an asynchronous discussion, which means that you can chime into the discussion anytime between 8am to 8pm. We will be moving on to another topic for each day therefore, if you have thoughts related to a specific module, please share them in the chat before 8PM .

---

2. Explain that you would like to learn about everyone as you are going to be working together for the next 16 days. You would also like to learn something special about each participant (fun facts etc). We suggest to share your name, major, campus and something special about themselves.
3. Begin by introducing yourself and share something interesting about yourself and ask participants to do the same.
    a. Some participants might be shy to share something special about themselves, be sure to give a lot of praise and encouragement for those who have responded to model support.

Yilamu Mfundzi Ngci Nami: A sexual harassment prevention intervention for University students in Eswatini © 2021 by Fortunate Shabalala, Anh Vo, Rebecca Fielding-Miller, Sakhile Masuku, Menelisi T.T. Dlamini
is licensed under Creative Commons Attribution-NonCommercial-NoDerivatives 4.0 International.
To view a copy of this license, visit https://creativecommons.org/licenses/by-nc-nd/4.0/

4. Because this takes place asynchronously, some participants might not respond right away. After a significant amount of people have introduced themselves (use your judgement). You can say that if anyone hasn't introduced themselves yet, they are welcome to chime in anytime through the discussion.
5. Explain to the group that with each module, we (including yourself) are learning through sharing out thoughts and opinions therefore it is useful for everyone to agree to some shared agreement ("shared ground rule") on how we should use this chat. Encourage everyone to suggest a rule to explain it to the group. Explain to the group that with each module, we are learning through sharing out thoughts and opinions therefore it is useful for everyone to agree to some shared agreement ("shared ground rule") on how we should use this chat. Encourage everyone to suggest a rule to explain it to the group. Participants should be encouraged to add on to each other's idea, reacts to previous messages that resonate with their expectation for a good learning environment and share their disagreement if they have any. Below, we included some ground rule suggestions to create a positive learning environment– if participants haven't brought it up, facilitators can also add to the discussion; or if someone have suggested these agreement already, as facilitator, you can ask the participants to expand, or add on to their thoughts.

Example exercise: One way to help facilitate this process is through this activity below- but feel free to use whatever strategies that work best for you from your experience. Participants are encouraged to start their sentences with the following.

Brainstorm session for this virtual learning environment.

1. I learn best when…
2. I am most likely to participate when…
3. Things that make my whole self feel welcome…
4. Things that make it difficult to learn collaboratively …

Suggested Shared Agreement for the discussion

1. Use respectful language and be kind to one another
2. Make space/ take space- Encourage shy participants to share their thoughts, and also on the flip end, not dominating the conversation
3. "I" statements and curious question-asking questions from a place of curiosity (rather than to judge or prove someone's wrong) . Talk about concerns and dilemmas to make the point that we all benefit from listening and understanding ourselves and others.
4. Keeping an open mind , rather than immediately judge the idea
5. Stories stay/ learnings leave- maintain confidentiality , please do not forward or screenshot other people's messages outside of this group
6. Stay within the focus of this group - please do not share videos, forwarded messages, or memes

Yilamu Mfundzi Ngci Nami: A sexual harassment prevention intervention for University students in Eswatini © 2021 by Fortunate Shabalala, Anh Vo, Rebecca Fielding-Miller, Sakhile Masuku, Menelisi T.T. Dlamini
is licensed under Creative Commons Attribution-NonCommercial-NoDerivatives 4.0 International.
To view a copy of this license, visit https://creativecommons.org/licenses/by-nc-nd/4.0/

7. Be present & care for yourself

---

6. Summarized the rule near the end of the day, around 7PM. Encourage group members to stick to these rules to ask them to remind you and one another if you or any of them err from them.

Key Takeaway

- Adhere to share agreement
- We learn from each other therefore, it is important for everyone to engage.

Yilamu Mfundzi Ngci Nami: A sexual harassment prevention intervention for University students in Eswatini © 2021 by Fortunate Shabalala, Anh Vo, Rebecca Fielding-Miller, Sakhile Masuku, Menelisi T.T. Dlamini
is licensed under Creative Commons Attribution-NonCommercial-NoDerivatives 4.0 International.
To view a copy of this license, visit https://creativecommons.org/licenses/by-nc-nd/4.0/

Instructions for Module 2-11:

1. Post the content and share the prompt.
2. Facilitate the conversation. Refer to our values when framing the guiding questions for the conversation. Use the guiding questions in the handbooks to guide the conversation. Keep in mind the key takeaways noted in each module.
    a. If you need assistance at any point in the discussion, please use the facilitator group chat to ask for help from other facilitators to resolve the situation.
3. Send the summary of the discussion at the end of the day at 11:30PM to the facilitator group.
4. Before starting the new module the next day, the U.S team will share back a graphic summarized key concepts that are being discussed across all the groups to facilitate sharing with participants reinforcing main ideas before the start of the next module.

Yilamu Mfundzi Ngci Nami: A sexual harassment prevention intervention for University students in Eswatini © 2021 by Fortunate Shabalala, Anh Vo, Rebecca Fielding-Miller, Sakhile Masuku, Menelisi T.T. Dlamini
is licensed under Creative Commons Attribution-NonCommercial-NoDerivatives 4.0 International.
To view a copy of this license, visit https://creativecommons.org/licenses/by-nc-nd/4.0/

Module 2: Let's Communicate

Learning Objectives: Recognize the importance of listening and communicating preferences.

Download Module Content

Prompts:

1. What factors can influence communication? What kinds of barriers might hinder effective communication? (probes for power dynamic)
2. What could be done to minimize barriers to communications?

Guiding Questions:

3. If you are Mimi or Msakhi, how could they respectfully resolve their disagreements?
4. What issues/problems come to mind when it comes to communicating?
5. How can we address these issues?

Key Takeaway

- When in doubt, just ask the person what they want
- The way in which the communication carried out matter (tone, frame, goals)

Yilamu Mfundzi Ngci Nami: A sexual harassment prevention intervention for University students in Eswatini © 2021 by Fortunate Shabalala, Anh Vo, Rebecca Fielding-Miller, Sakhile Masuku, Menelisi T.T. Dlamini
is licensed under Creative Commons Attribution-NonCommercial-NoDerivatives 4.0 International.
To view a copy of this license, visit https://creativecommons.org/licenses/by-nc-nd/4.0/

Module 3: Consent

Learning Objective: Explain how ongoing consent is part of a healthy relationship.

Download Module Content

Prompts:

1. At what point is consent needed?
2. Can you revoke consent at any point?

Guiding Questions:

3. What is consent?
4. If you were Pepe or Llyana in this story, what would make you feel respected?
5. How do these two people are feeling in this scenario?
6. Who is the right person to ask consent from?
7. What is the fair outcome for both Pepe and Llyana?
8. Thinking about Pepe's situation, Llyana has paid for the date, what is the power dynamic at play in this scenario?

Key Takeaways:

- It is important to ask for consent
- Consent can always be revoked after interaction starts

Related Resources:

- Consent and Tea Youtube

Yilamu Mfundzi Ngci Nami: A sexual harassment prevention intervention for University students in Eswatini © 2021 by Fortunate Shabalala, Anh Vo, Rebecca Fielding-Miller, Sakhile Masuku, Menelisi T.T. Dlamini
is licensed under Creative Commons Attribution-NonCommercial-NoDerivatives 4.0 International.
To view a copy of this license, visit https://creativecommons.org/licenses/by-nc-nd/4.0/

Module 4: Gender Based Violence Spectrum

Learning Objective:

Describe the process of how normalization of harassment is part of explicit violence

Download Module Content

Prompts:

1. How can normalizing the things at the bottom of the pyramid lead to explicit violence?
2. How does the spectrum of rape culture pyramid reinforce gendered power hierarchies that constraints women, men and sexual/gender minority?

Guiding Questions:

1. Those who have experienced catcalling, how does that make you feel?
2. For those who haven't experienced catcalling, what do you notice/think when hearing about this?
3. How does catcalling function within this pyramid? What power dynamic is at play?
4. How does the spectrum of GBV reinforce gendered power hierarchies that constrain women?
    a. How does unequal pay relate to sexual violence against women?
5. How does the spectrum of GBV reinforce gendered power hierarchies that constrain men?
    a. How does a comment such as "boys will be boys" affect men?
6. How does the spectrum of GBV reinforce gendered power hierarchies that contrain sexual/gender minority?

Key takeaways:

- Harassment is about power and control, not about attraction
- The attitudes and actions on the bottom tiers (normalization) reinforce, excuse and drive those higher up (explicit violence)
- Violence is not an isolated incidents but rather a continuum
- To combat harassment, we have to acknowledge every attitude and practices that perpetuate these elicit violence.

Yilamu Mfundzi Ngci Nami: A sexual harassment prevention intervention for University students in Eswatini © 2021 by Fortunate Shabalala, Anh Vo, Rebecca Fielding-Miller, Sakhile Masuku, Menelisi T.T. Dlamini
is licensed under Creative Commons Attribution-NonCommercial-NoDerivatives 4.0 International.
To view a copy of this license, visit https://creativecommons.org/licenses/by-nc-nd/4.0/

Module 5: Reporting

Learning Objective: Understand how to report incident of harassment through official channel

Download Module Content

Prompt Question:

1. If you have a friend who needed support after experiencing harassment, what mechanism of reporting are helpful for you or your friends?
2. What component of the policy do you think could be improve on?
3. What are some questions you have about the policy?

Note: Facilitators do not have to answer these questions but rather noting them down for the next module.

Yilamu Mfundzi Ngci Nami: A sexual harassment prevention intervention for University students in Eswatini © 2021 by Fortunate Shabalala, Anh Vo, Rebecca Fielding-Miller, Sakhile Masuku, Menelisi T.T. Dlamini
is licensed under Creative Commons Attribution-NonCommercial-NoDerivatives 4.0 International.
To view a copy of this license, visit https://creativecommons.org/licenses/by-nc-nd/4.0/

Module 6: Imagine

Learning Objective: Envision a future where support is prioritized over punishment for people who experience violence and people who perpetrate violence

Download Module Content

Prompt Question:

1. In a perfect world, how should we provide support for survivors of violence?

Guiding Questions:

1. Given our previous module on reporting, what component of the policy do you think could be improve on?
2. What kind of campus life can you imagine in UNESWA
3. How would you like the current system to change?
4. How do you feel about the current justice system where punishment is the main goal? What would you change about it
5. In a perfect world, how should we respond to people who perpetrated violence? Does it stop at punishment or does it require something more?
    a. How would you want to be approached for your actions if you've hurt somebody

General Probing Questions:

1. Can you provide an example?
2. What does this look like?

Resource:
https://thetyee.ca/Citizentoolkit/2004/11/15/CanImagSaveUs/

Yilamu Mfundzi Ngci Nami: A sexual harassment prevention intervention for University students in Eswatini © 2021 by Fortunate Shabalala, Anh Vo, Rebecca Fielding-Miller, Sakhile Masuku, Menelisi T.T. Dlamini
is licensed under Creative Commons Attribution-NonCommercial-NoDerivatives 4.0 International.
To view a copy of this license, visit https://creativecommons.org/licenses/by-nc-nd/4.0/

Module 7: Supporting a friend

Learning Objective: How to respond to someone who has experienced harassment/violence when they disclose their experiences and provide support in a trauma-informed manner.

Download Module Content

Prompt Question:

1. How do you support a friend who experiences violence in a way that is respectful (respect for choices, wishes, rights , and dignity) ?
2. How to take care of yourself while supporting a friend?

Guiding Questions:

1. How do you want a friend to support you when you feel helpless?
2. How does consent play a role in supporting survivors?
3. What are some ways you can empower a survivor of sexual harrassment or assault?
    a. What are some ways you can support survivors moving forward?

Key takeaways:

- Making sure the person who experienced violence are in control and let them make their own choices
- Ask the person who experienced violence for informed consent before connecting them to anyone else.
- Safety is a priority and ensure that their story are confidential (unless given consent)
- Ask for permission before seeking support on their behalf
- Avoid asking for the details outside of what the person who experienced violence already share (they can choose how much or little they like to share- if at all)

Resources:
https://www.icrc.org/en/document/supporting-victims-survivors-sexual-violence

Yilamu Mfundzi Ngci Nami: A sexual harassment prevention intervention for University students in Eswatini © 2021 by Fortunate Shabalala, Anh Vo, Rebecca Fielding-Miller, Sakhile Masuku, Menelisi T.T. Dlamini
is licensed under Creative Commons Attribution-NonCommercial-NoDerivatives 4.0 International.
To view a copy of this license, visit https://creativecommons.org/licenses/by-nc-nd/4.0/

Module 8: Deconstructing Rape Myths

Learning Objective: Understand common myths about sexual harassment and how they contribute to victim blaming culture.

Download Module Content

Prompt Question:

1. Thinking about the rape culture pyramid in Module 4, where do these statements fall on the pyramid? (probe for the connection between normalization to explicit violence)
2. How can these perceptions potentially lead to self-blame?

Guiding Questions:

1. Imagine being in one of these scenarios from the graph and all your friends side with the perpetrator. How would you feel?

Key takeaways:

- Rape myths are the building blocks of explicit violence
- Rape myths are pervasive but are not true

Yilamu Mfundzi Ngci Nami: A sexual harassment prevention intervention for University students in Eswatini © 2021 by Fortunate Shabalala, Anh Vo, Rebecca Fielding-Miller, Sakhile Masuku, Menelisi T.T. Dlamini
is licensed under Creative Commons Attribution-NonCommercial-NoDerivatives 4.0 International.
To view a copy of this license, visit https://creativecommons.org/licenses/by-nc-nd/4.0/

Module 9: Gender/Sexual Minorities

Learning Objective: Recognize discriminatory and harmful experiences among LGBTQIA+.

Download Module Content

Content: The content for this module is an excerpt from the poem On Coming Out by Lee Mokobe.

Prompt Question:

1. What can we do to ensure people who are sexual minority can feel safer on campus?

Guiding Questions:

1. What did you think of this poem?
2. Lee Mokobe talked about his experience with harassment at school in this poem, what can we do to make sure transgender and other sexual/gender minority feel safer? (probe for what can be done at the individual, community, university, and system levels)
3. Can you provide an example that you've seen of support for lgbtq that you think should be copied?

Key takeaways: Gender and sexual minorities should be treated with respect and need support

Yilamu Mfundzi Ngci Nami: A sexual harassment prevention intervention for University students in Eswatini © 2021 by Fortunate Shabalala, Anh Vo, Rebecca Fielding-Miller, Sakhile Masuku, Menelisi T.T. Dlamini
is licensed under Creative Commons Attribution-NonCommercial-NoDerivatives 4.0 International.
To view a copy of this license, visit https://creativecommons.org/licenses/by-nc-nd/4.0/

Module 10: Commitment to Ending Gender Based Violence

Learning Objective: Create a commitment about ending gender based violence

Download Module Content

Prompt Question:

Share a commitment that you want to make to yourself/our groups about ending gender based violence. Explain

Key takeaways: Ending sexual harrassment requires systemic change brought about by advocacy
Share a #, tag SNASH, share this information at the end of days

---

Resources to share with students

- https://www.11thprincipleconsent.org/consent-propaganda/share-model-for-informed-enthusiastic-consent/

Yilamu Mfundzi Ngci Nami: A sexual harassment prevention intervention for University students in Eswatini © 2021 by Fortunate Shabalala, Anh Vo, Rebecca Fielding-Miller, Sakhile Masuku, Menelisi T.T. Dlamini
is licensed under Creative Commons Attribution-NonCommercial-NoDerivatives 4.0 International.
To view a copy of this license, visit https://creativecommons.org/licenses/by-nc-nd/4.0/
